# Mutational spectrum of *APC *and genotype-phenotype correlations in Greek FAP patients

**DOI:** 10.1186/1471-2407-10-389

**Published:** 2010-07-22

**Authors:** Florentia Fostira, Georgia Thodi, Raphael Sandaltzopoulos, George Fountzilas, Drakoulis Yannoukakos

**Affiliations:** 1 Molecular Diagnostics Laboratory, I/R-RP, National Center of Scientific Research "Demokritos", Athens, Greece; 2 Laboratory of Gene Expression, Molecular Diagnosis and Modern Therapeutics, Department of Molecular Biology and Genetics, Democritus University of Thrace, Alexandroupolis, Greece; 3 Department of Medical Oncology, Papageorgiou Hospital, Aristotle University of Thessaloniki School of Medicine, Thessaloniki, Greece; 4 Hellenic Cooperative Oncology Group, Athens, Greece

## Abstract

**Background:**

Familial adenomatous polyposis, an autosomal dominant inherited disease caused by germline mutations within the *APC *gene, is characterized by early onset colorectal cancer as a consequence of the intrinsic phenotypic feature of multiple colorectal adenomatic polyps. The genetic investigation of Greek adenomatous polyposis families was performed in respects to *APC *and *MUTYH *germline mutations. Additionally, all available published mutations were considered in order to define the *APC *mutation spectrum in Greece.

**Methods:**

A cohort of 25 unrelated adenomatous polyposis families of Greek origin has been selected. Genetic testing included direct sequencing of *APC *and *MUTYH *genes. *APC *gene was also checked for large genomic rearrangements by MLPA.

**Results:**

Analysis of the *APC *gene performed in a Greek cohort of twenty five FAP families revealed eighteen different germline mutations in twenty families (80%), four of which novel. Mutations were scattered between exon 3 and codon 1503 of exon 15, while no large genomic rearrangements were identified.

**Conclusion:**

This concise report describes the spectrum of all *APC *mutations identified in Greek FAP families, including four novel mutations. It is concluded that the Greek population is characterized by genetic heterogeneity, low incidence of genomic rearrangements in *APC *gene and lack of founder mutation in FAP syndrome.

## Background

The precancerous syndrome familial adenomatous polyposis (FAP) is transmitted as an autosomal dominant trait and is caused by germline mutations within the *adenomatous polyposis coli (APC) *gene [1,2]. FAP syndrome is primarily characterized by the presence of multiple colorectal adenomatous polyps and a variable range of extracolonic manifestations [3].

The classical FAP phenotype involves the presence of at least 100 colorectal adenomas developed at a young age, whereas the profuse phenotype is associated with the presence of thousands polyps and an increased risk of developing very early onset colorectal cancer. The discrete location of mutations on the *APC *gene determines the particular FAP phenotypic manifestations, as well as the severity of the disease [4,5]. Notably, a particular severe phenotype, involving a higher number of polyps and an earlier onset of colorectal cancer, has been observed in patients carrying mutations in the most mutated codon (codon 1309) of the *APC *gene [6-8], with a population frequency ranging from 0% to 29% [9]. Additionally, a study in the Balearic Island revealed that the hotspot c.3183_3187delACAAA mutation has a founder effect [10]. This study is the only report of a founder effect in FAP syndrome, since the *APC *gene is characterized by a high rate of *de novo *mutations, enhancing the spectrum of mutations identified [9,11,12].

On the other hand, an attenuated form of disease, characterized by the later onset and smaller number of polyps, is caused by mutations on the extreme 5' end or the 3' end [13] or the alternatively spliced region of exon 9 of the gene [14-16]. In the latter case, the mutated codon can be ruled out during the normal splicing occurring in colonic mucosa, resulting in a milder phenotype. The alternative splicing mechanism involving exon 9, where removal of codons 312 to 412 is taking place, produces a shorter *APC *isoform. Both isoforms are present in normal tissues, the expression of which is tissue-specific, with the full version being more abundant in most tissues [1].

Gardner syndrome, constituting a phenotypic variant of FAP is defined by the association of colorectal polyposis and the clinical triad of desmoid tumours, osteomas and epidermoid cysts, appears to be associated with a classic form of FAP syndrome [17]. These patients are characterized by high mortality rates due to obstruction and perforation of surrounding structures within the bowel, caused by desmoids tumours [18].

The last few years a new type of polyposis syndrome, resembling to the attenuated form of FAP, has been identified. The so-called *MUTYH *associated polyposis syndrome (MAP), which is inherited in an autosomal recessive manner, is caused by mutations of the base excision repair gene *MUTYH *[19].

In this study the *APC *coding region has been analyzed for point mutations and large deletions in 25 families matching the FAP syndrome criteria. Eighteen different mutations, out of which four novel, have been identified in twenty families. *APC *negative families were then checked for germline *MUTYH *mutations. The aim of this study is to describe the complete *APC *mutation spectrum identified in Greek FAP families and to correlate it to the phenotype.

## Methods

### Patients

FAP patients and their relatives were referred through the Molecular Diagnostics Laboratory of N.C.S.R Demokritos of Athens. The specific study focuses on 42 individuals from 25 families. Patients with clinical detection of colorectal polyps, ranging from 50 to 1000 adenomas, were included in the study. In order for a detailed family tree to be constructed, an interview is taking place with as many family members as possible and informed consent was taken from all patients before genetic testing. The study was approved by the Bioethics Committee of the National Centre for Scientific Research "Demokritos" (Reference Number 240/EHΔ/10.8) in agreement with the 1975 Helsinki statement, revised in 1983.

### DNA and RNA isolation

Total genomic DNA was isolated from peripheral blood leukocytes following the salt extraction procedure [20]. Total RNA was extracted from blood using Trizol (Invitrogen, Paisley, UK) following standard protocols.

### Reverse Transcription PCR

A template-primer mix containing 1000 ng of total RNA and 60 μM of random hexamers were boiled at 65°C for 10 minutes, followed by immediate cool on ice. After the addition of 8 mM MgCl_2_, 1 mM dNTPs, 5 mM DTT, 20 U RNase inhibitor and 10 U MMLV reverse transcriptase (Roche Diagnostics, Mannheim, Germany) the mixture was incubated at 50°C for 30 minutes followed by enzyme inactivation at 85°C for 5 min. cDNA were used for subsequent PCR amplification. Primers used are available on request.

### Mutational analysis

The complete coding sequence of the *APC *gene, comprising of 15 exons, as well as all 16 exons of the *MUTYH *gene, including splice junctions, were amplified by Polymerase Chain Reaction. PCR conditions and primers used are available from the authors upon request. PCR product purification was performed using a vacuum driven ultrafiltration purification system, where PCR samples are transferred to a filter plate with a membrane resin, which retains the PCR products free from non-incorporated nucleotides and primers (Macherey-Nagel, Düren, Germany). Sequencing reactions were performed using the v.3.1 BigDye Terminator Cycle Sequencing kit (Applied Biosystems, Foster City, CA) and PCR products were electrophorized on the ABI Prism^® ^310 Genetic Analyzer. Sequences obtained were aligned, using Sequencher^® ^PC software (Gene Codes, USA), with reference sequences from Genbank (NM_000038) and examined for the presence of mutations.

### Multiplex ligation-dependent probe amplification (MLPA)

For the detection of large deletions or duplications of the *APC *gene, Multiplex Ligation-dependent Probe Amplification (MLPA) was carried out using the SALSA P043 APC exon deletion test kit (MRC-Holland, Netherlands), following manufacturer's instructions.

## Results

**Figure 1:**
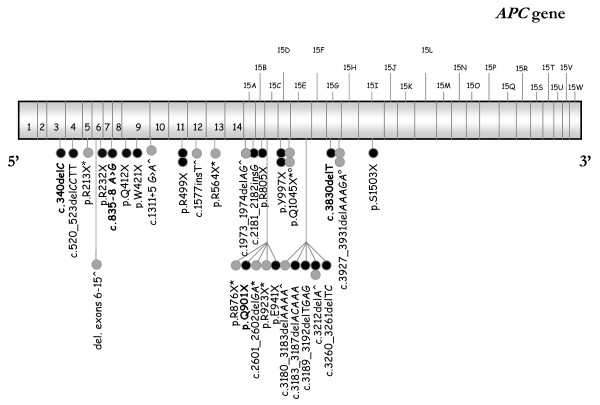
**The mutational spectrum of *APC *germline mutations identified in 35 unrelated Greek families**. Each bullet represents a carrier family of an *APC *germline mutation. Black bullets represent mutations identified by our group, whereas grey bullets represent mutations identified by others in Greek families. * [29], ° [30], ^ [31]

In the present study 18 different *APC *germline mutations have been identified in 20 out of the 25 Greek families screened, suggesting a detection rate of 80%. Four mutations are described here for the first time, to the extent of our knowledge, whereas two *de novo *mutations have been found, suggesting a 10% frequency in the studied population. Eight mutations were nonsense, caused by a single base substitution; one was an intronic base substitution causing aberrant splicing, one caused aberrant splicing in the alternatively spliced region, located in exon 9 of the *APC *gene [21], while the remaining eight were either insertions or deletions causing a frameshift and the introduction of a premature stop codon. The distribution of germline mutations identified, occurring over the 5' half of the *APC *gene, is in concert with most reports, while quite interesting is the low frequency of the two hotspot mutations (codons 1061 and 1309) identified in most populations studied. Despite the extensive analysis of all samples for large deletions or rearrangements, using the commercially available MLPA kit, none was detected within our cohort. The *APC *mutational spectrum, which includes the findings of the specific study, as well as previously reported mutations in the Greek population are summarized in Figure 1, whereas all patient clinical data, family members tested and detailed mutation information are summarized in Table 1.

**Table 1:** *APC *germline mutations identified in Greek FAP patients with supportive clinical data

Family ID	Exon	Mutation	Consequence	Age at diagnosis	Phenotype	Cancer	Reason for diagnosis
**F1195**	**3**	**c.340delC**	**p.Pro114LeufsX11**	**49**	**50-100 colorectal polyps**	**N**	symptoms

F1275	4	c.520_523delCCTT	p.Pro174X1	30	50-100 colorectal polyps	N	family member

F75	6	c.694C > T	p.Arg232X	37	50-100 colorectal polyps	N	symptoms

**F1270**	**8**	**c.835-8 A > G**	**aberrant splicing of ex. 7**	**38**	**100-200 colorectal polyps**	**N**	**symptoms**

F76	9	c.1234 C > T	p.Gln412X & aberrant splicing of ex. 9	30	30-50 colorectal polyps	N	symptoms

F1196	9	c.1263G > A	p.Trp421X	28	100-1000 colorectal polyps, UGI polyps, desmoids	Y	symptoms

F113	11	c.1495C > T	p.Arg499X	51	100-1000 colorectal polyps	N	symptoms

F741	11	c.1495C > T	p.Arg499X	40	100-200 colorectal polyps	N	symptoms

F880	15A	c.2181_2182insG	p.Asn728GlufsX6	26	100-1000 colorectal polyps	N	family member +symptoms

F160	15B	c.2413 C > T	p.Arg805X	23	100-1000 colorectal polyps, desmoids, thyroid cancer	Y	family member

**F1186**	**15C**	**c.2701C > T**	**p.Gln901X**	**28**	**100-1000 colorectal polyps**	**Y**	**family member +symptoms**

F899	15D	c.2821G > T	p.Glu941X	23	50-100 colorectal polyps	N	family

F274	15D	c.2991 T > A	p.Tyr997X	56	100-1000 colorectal polyps	N	symptoms

F474	15D	c.2991 T > A	p.Tyr997X	26	100-1000 colorectal polyps	N	family member

F83	15E	c.3183_3187delACAAA	p.Gln1062FsX1	36	100-1000 colorectal polyps	Y	symptoms

F446	15E	c.3189_3192delTGAG	p.Glu1064LysfsX61	34	100-1000 colorectal polyps	N	family member

F71	15E	c.3214delA	p.Ser1072ValfsX54	35	100-1000 colorectal polyps	N	symptoms

F50	15E	c.3260_3261delTC	p.Leu1087GlnfsX31	23	100-1000 colorectal polyps	N	symptoms

**F85**	**15G**	**c.3830delT**	**p.Leu1277TyrfsX11**	**28**	**> 1000 colorectal polyps, 150 UGI polyps**	**N**	**family member +symptoms**

F153	15I	c.4508C > G	p.Ser1503X	48	100-200 colorectal polyps	N	symptoms

cDNA numbering is based on reference sequence: GenBank NM_000038. +1 corresponds to the A of the ATG translation initiation codon. Novel mutations are highlighted in **boldface**.

### Identification and characterization of families with novel mutations

The four novel mutations identified were scattered along the Greek mutation spectrum characterized by this study, with one located on exon 3, one on intron 7 and two within exon 15. Proband 1195, who was classified as an attenuated FAP case, was found to carry a novel mutation (c.340delC), located on codon 114 of exon 3. The specific mutation is possibly *de novo*, since both proband's parents deceased at their late seventies, with no apparent FAP clinical symptoms; no genetic material of the parents was available.

Family 1186 is classified as a classic FAP phenotype case, in the absence of extracolonic manifestations. Direct sequencing of the *APC *gene revealed the nonsense mutation c.2701 C > T, located on exon 15. The proband's mother was diagnosed with colorectal cancer at the age of 30 in the presence of multiple colorectal polyps, while her brother, who was asymptomatic at the age of 30, did not carry the mutation.

Another novel mutation (c.3830delT) identified in this study involves a family (F85) characterized by the severe form of FAP. The proband's mother was diagnosed with colorectal cancer and deceased at the age of 37, while one of her sister and one of her brother proceeded in having a total colectomy at the ages of 20 and 25, respectively, due to the existence of thousands polyps throughout their colon and rectum. The region encoding for the essential 20-amino acid repeats, where β-catenin binding is taking place, involves codons 1265 to 2065. Therefore the particular alteration disrupts most 20-amino acid repeats, possibly explaining the severity of the family's phenotype.

**Figure 2:**
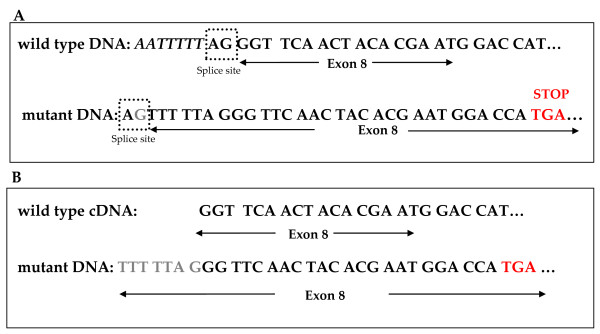
**Characterization of the intonic base substitution causing alternative splicing**. **(A) **DNA sequence of intron 7-exon 8 boundaries, where the AG acceptor site is highlighted in the dotted box in both the wild type and the mutant DNA and the premature termination codon is coloured in red. **(B) **The 7-bp insertion upstream of exon 8 on cDNA from the patient is coloured in grey.

Direct sequencing of DNA extracted from patient 1270 revealed a single base substitution located in intron 7, eight nucleotides upstream of exon 8 (c.835-8 A > G). This change results in a 7-bases insertion, which was detected by sequencing of the corresponding cDNA fragment. This is taking place due to the creation of a new AG splice acceptor site seven bases upstream the actual site, causing a frameshift and a premature termination codon. A schematic representation is shown on Figure 2.

### Family with multiple phenotypic features

A particular aggressive phenotypic expression of the mutation c.1263 G > A, located on exon 9, was identified in a 28-year old female patient. The proband was diagnosed with multiple adenomatous polyps, mostly allocated in the sigmoid and transverse colon, as well as gastric polyps of adenoma nature at the age of 28. She undertook a total colectomy and performed surgical removal of gastric polyps twice, at the age of 28 and 34. At her 34 years of age she developed a desmoid tumour located at the mesenterium, while at her 37 was diagnosed with an additional desmoid tumour situated between the endometrium and the urinary tract. It is hypothesized that the mutation was maternally inherited, since the proband's mother and maternal uncle deceased from generalized cancer at the age of 52 and 56, respectively and therefore, there is no available genetic material to confirm this. The proband's daughter, aged 12 years old, was tested negative for the specific mutation. This family could be possible a case of Gardner syndrome, although the characteristic osteomas and epidermoid cysts were not possible to be verified clinically, as the proband deceased soon after the genetic diagnosis.

### Genotype-Phenotype correlation

Three patients in our cohort were found to carry mutations in the *APC *regions that have been associated with the attenuated FAP phenotype. More specifically, the mutations were located in exons 3, 4 and the alternatively spliced region of exon 9 of *APC *gene. All three patients showed the attenuated FAP phenotype, characterized by low polyp burden, ranging from 30 to 100 adenomas, in the absence of exctracolonic manifestations. Moreover, none of the aforementioned patients developed colorectal cancer.

Furthermore, twelve patients were characterized by the classical FAP phenotype. These patients were found to carry mutations between codons 421 to 1503. Colorectal cancer was diagnosed in four cases, with a mean age at onset of 28.7 years. The number of polyps ranged from 100 to 1000. Desmoids were reported in two patients, while thyroid cancer has been described in one case.

Remarkably, a relatively frequent mutation (p.R232X) located in exon 6 of the gene, mostly associated with the classical FAP phenotype, was identified in a patient who was presented with an attenuated phenotype. Moreover, three more patients carrying mutations in gene regions associated with the classic FAP phenotype (F1270: intron 7, F899: exon 15 and F153: exon 15) were presented with a medium polyp burden, ranging from 100-200 polyps.

A patient carrying the novel mutation c.3830delT (p.Leu1277TyrfsX11) was presented with the severe form of FAP, characterized by profuse colorectal polyposis, as well as upper gastrointestinal polyposis.

### APC- negative families

Four out of the five *APC*- mutation negative patients show an attenuated phenotype, comparably late age of diagnosis and low polyp burden. The fifth is presented with a number of colorectal adenomatous polyps (> 100) compatible to FAP syndrome and colorectal cancer in two family relatives around the age of 35. All *APC*- mutation negative patients have been tested for *MUTYH *germline mutations. No *MUTYH *mutations were identified in these families. Furthermore, no *APC *deletions or rearrangements have been detected by MLPA.

### Lack of founder effect

As indicated in Figure 1, the majority of mutations identified in our cohort were unique, except for two, which were shared between two families. Even if we combine all available data regarding *APC *mutations in Greek families, only three more mutations are recurrent (identified also in two families) in a total of thirty different mutations. These data are consistent with the lack of a founder effect in respects to FAP syndrome in the Greek population.

### Frequency of rearrangements and the "classic" FAP mutations

Despite the fact that the MLPA technique has been introduced in our series of experiments, no large rearrangements within the *APC *gene have been identified in our cohort. Interestingly, only one such mutation has been reported in the Greek population, suggesting a frequency rate of 2.8%. Another interesting finding is the frequency of the two mutational hotspots (codon 1061: c.3183_3187delACAAA and codon 1309: c.3927_3931delAAAGA) in the Greek population. The frequent 1061 mutation has been identified once in our cohort (frequency rate of 2.8%), while the c.3927_3931delAAAGA mutation located on codon 1309 has been reported twice in the total Greek mutational spectrum, suggesting a frequency rate of 5.7%.

## Discussion

Multiple genetic studies performed in FAP syndrome have shown a quite clear pattern of phenotype-genotype correlation [5,22]. This finding is consistent within our cohort, and constitutes a great tool in the early mutation identification and the correct clinical management of *APC *mutation carriers, as well as their family relatives. The Greek *APC *mutation spectrum is characterized by heterogeneity, while it is notable that almost every FAP family carries a different mutation. Moreover, four novel mutations have been identified, one of which causes attenuated FAP. One novel single base deletion (c.3830delT), causing a frameshift and a termination codon was found to be associated with the severe form of FAP syndrome. This finding is in concert with already published data, highlighting the association of profuse polyposis to mutations located between codons 1250 and 1464 of *APC *[4].

An interesting case reported within our cohort is the one carrying the Gardner's syndrome characteristics. Although the characteristic osteomas and epidermoid cysts could not be confirmed on our patient, the fact that the specific mutation, c.1263 G > A (p.W421X), has been described in a large Spanish family with typical Gardner's syndrome features [23], indicate that our family can be classified as a Gardner syndrome case. Surprisingly, the proband in the Spanish family did not develop desmoids tumours. Therefore, patients carrying the exact same mutation are presented with a quite different phenotypic expression, which is clearly influenced by modifier genes and/or environmental factors.

A 20% of FAP families in our study were tested negative for point mutations and rearrangements of the entire *APC *coding sequence, as well as for germline *MUTYH *mutations. A quite frequent cause of FAP has been illustrated to be the allele-specific reduced *APC *expression in patients with unidentified *APC *mutation [24]. A restriction of this technique includes the requirement for patient heterozygosity in at least one nucleotide polymorphism within the *APC *coding region. Unfortunately, this technique could not be performed in our patients since the genetic material (RNA) needed was not available in a subset of patients, whereas in the two *APC*-negative, where RNA was at hand, no heterozygosity at coding polymorphisms has been identified. Finally, the existence of genes, other than *APC *and *MUTYH*, susceptible to colorectal adenomas and cancer cannot be excluded.

The surprising findings of the specific work, which reviews all the reported *APC *mutations identified within the Greek population, is the low frequency of gene rearrangements, as well as the rare presence of the common reported mutations at codons 1061 and 1309. In most populations examined, large gene rearrangements contribute to the 5% of total mutations identified [9], whereas in specific populations, such as the Swedish, this may rise up to 9% [25]. The Greek adenomatous polyposis cohort, constituting of forty families in total, shows a frequency of 2.8%, a rate lower than the expected. Additionally, the rate of the two highly frequent mutations, c.3183_3187delACAAA and c.3927_3931delAAAGA, in the Greek population was found to be 2.8% and 5.7%, respectively. This is an interesting finding, as these two mutations are usually found in higher frequencies in most populations [26].

The low detection rate for the two hot spot mutations, although rare, has been previously reported in the Galician population, where the 1061 mutation was not detected at all and the 1309 mutation was detected in a frequency of 5.2% [27]. Furthermore, the common mutation at codon 1309 was not identified in a series of Australian FAP families [28]. This observation, along with the absence of a founder effect in *APC*, highlights the mutation heterogeneity, probably caused by the high emigration rates, within the Greek adenomatous polyposis patients.

According to the literature, attenuated FAP characterizes families carrying mutations on the 5'end of the gene, the alternatively spliced region of exon 9 and the 3' end of the gene to codon 1580. Severe FAP is associated with mutations in codon 1309, while classic FAP mutations span the 5' end of exon 15. These observations are in consistency with the findings in the Greek population, as well.

## Conclusion

This study reports the *APC *mutation spectrum identified in Greek FAP families, including four novel mutations. Furthermore, the genetic heterogeneity of the Greek population, characterized by low incidence of genomic rearrangements in the *APC *gene can be a useful tool in the genetic testing available for the predictive diagnosis of at risk family members, followed by the customized clinical management.

## Abbreviations

*APC*: adenomatous polyposis coli; FAP: familial adenomatous polyposis; *MUTYH*: human homologue of *MUT-Y*

## Competing interests

The authors declare that they have no competing interests.

## Authors' contributions

FF participated in the design of the study, carried out the experimental procedures and wrote the manuscript. GT carried out the MLPA experiments. RS participated in its design and corrected the manuscript. GF participated in the coordination of the study. DG conceived and coordinated the study. All authors read and approved the final manuscript.

## Pre-publication history

The pre-publication history for this paper can be accessed here:

http://www.biomedcentral.com/1471-2407/10/389/prepub
